# Incidence and Spatial distribution of Human and Livestock Anthrax in Cao Bang Province, Vietnam (2004–2020)

**DOI:** 10.1089/vbz.2022.0072

**Published:** 2023-05-12

**Authors:** Tan Luong, Thi Bach Be, Minh Dat Hoang, Thi Thu Ha Hoang, Quang Thai Pham, Thi Mai Hung Tran, Hoang Dung Ho, Pham Thanh Long, Jason K. Blackburn

**Affiliations:** ^1^ Spatial Epidemiology and Ecology Research Laboratory, Department of Geography, University of Florida, Gainesville, Florida, USA.; ^2^ Emerging Pathogens Institute, University of Florida, Gainesville, Florida, USA.; ^3^ National Institute of Hygiene and Epidemiology, Hanoi, Vietnam.; ^4^ Cao Bang Provincial Center for Disease Control, Cao Bang City, Cao Bang, Vietnam.; ^5^ Cao Bang Provincial Sub-Department of Plantation and Animal Husbandry, Cao Bang City, Cao Bang, Vietnam.; ^6^ School of Preventive medicine and public health, Hanoi Medical University, Hanoi, Vietnam.; ^7^ Department of Animal Health, Ministry of Agriculture and Rural Development, Hanoi, Vietnam.

**Keywords:** spatial analysis, human anthrax, livestock anthrax, Cao Bang, Vietnam

## Abstract

Specific knowledge on the distribution of anthrax, a zoonosis caused by *Bacillus anthracis*, in Southeast Asia, including Vietnam, remains limited. In this study, we describe disease incidence and spatial distribution of human and livestock anthrax using spatially smoothed cumulative incidence from 2004 to 2020 in Cao Bang province, Vietnam. We employed the zonal statistics routine a geographic information system (GIS) using QGIS, and spatial rate smoothing using spatial Bayes smoothing in GeoDa. Results showed higher incidence of livestock anthrax compared with human anthrax. We also identified co-occurrence of anthrax in humans and livestock in northwestern districts and the province center. Livestock anthrax vaccine coverage was <6% and not equally distributed among the districts of Cao Bang province. We provide implications for future studies and recommend improving disease surveillance and response through data sharing between human and animal health sectors.

## Introduction

Anthrax is a zoonosis caused by *Bacillus anthracis* and primarily reported in herbivorous animals. *Bacillus anthracis* infects humans commonly through handling sick animals and consuming contaminated animal products (WHO, 2008). The disease is vaccine-preventable; vaccine coverage in livestock must be sufficiently maintained to reduce livestock disease burden and subsequently in humans (Kracalik et al., 2017). The northern provinces of Vietnam share a border with districts in China, which reported high incidence of anthrax in humans and livestock (Chen et al., 2016).

Active livestock trade in this area would increase the risk of disease transmission across the border (Oyetola et al., 2021). Specific knowledge regarding anthrax in Southeast Asia, including Vietnam, remains limited. In this study, we describe the incidence of human and livestock anthrax for Cao Bang province in northeastern Vietnam from 2004 to 2020 and compare the incidence with livestock anthrax vaccine coverage from 2014 to 2020. We also estimate the spatial distribution using smoothed human and livestock anthrax incidence from 2004 to 2020.

## Materials and Methods

**FIG. 1.**
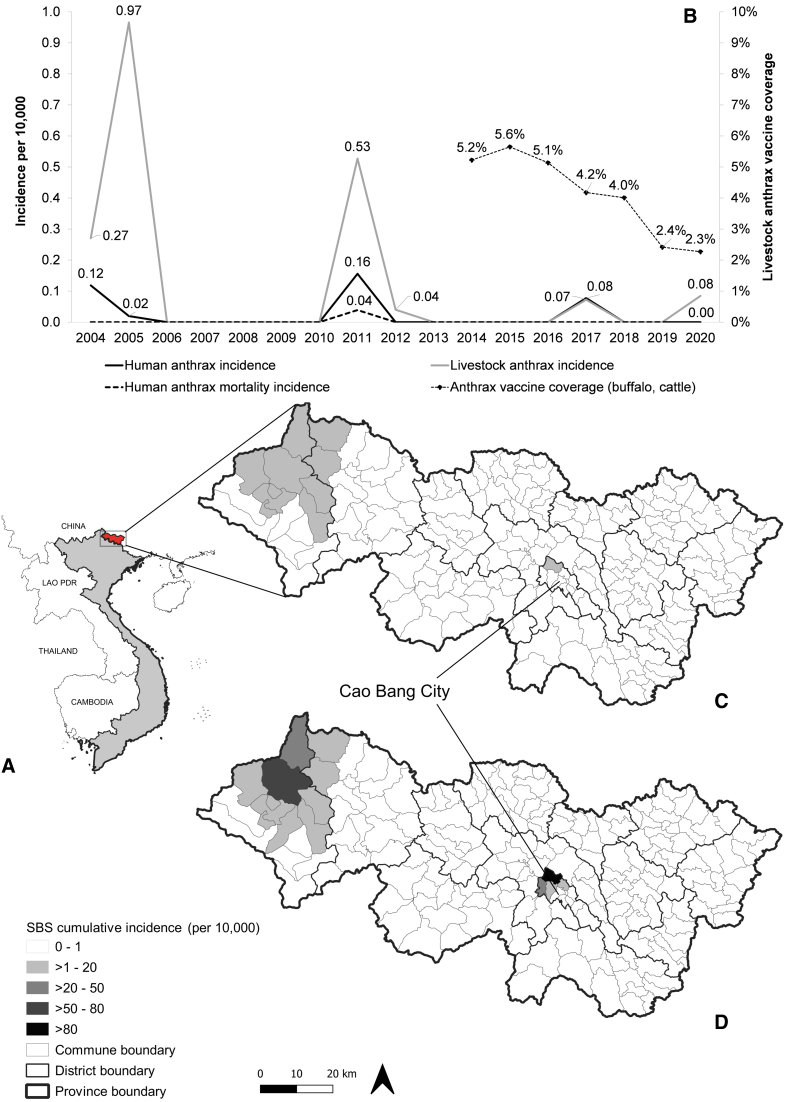
Temporal and spatial distribution of anthrax in humans and livestock in Cao Bang province, Vietnam, 2004–2020. **(A)** Cao Bang province in the Northeastern Vietnam. **(B)** Provincial-level incidence of human anthrax, livestock anthrax, human anthrax fatality (per 10,000) from 2004 to 2020, and anthrax vaccine coverage in buffalo and cattle from 2014 to 2020. Spatial distribution of anthrax using Spatial Bayes Smoothed (SBS) cumulative incidence of anthrax (per 10,000) in humans **(C)** and livestock **(D)**. The SBS cumulative incidence was constructed in GeoDa version 1.20.

Cao Bang is a mountainous province in northernmost Vietnam (Fig. 1A). For this study, we compiled anthrax cases from Cao Bang provincial disease reporting systems for the human and veterinary health sectors from 2004 to 2020. The number of livestock anthrax vaccine doses at provincial and district levels were also provided for each year from 2014 to 2020 (Supplementary Data S1).

Commune-level (subdistrict) human population was estimated annually from 2004 to 2020 the using zonal statistics routine in a geographic information system (GIS) using QGIS v3.24 and gridded unconstrained UN adjusted population counts from WorldPop. Commune-level livestock population estimates were also derived from zonal statistics using global data for buffalo, cattle, and goats in 2010 (Gilbert et al., 2018) (Supplementary Data S1). In addition, a district-level data set for livestock population (buffalo, cattle, and goats) from 2010 to 2020 was provided by local animal health administration and we estimated livestock population for each year from 2004 to 2009 based on growth rates from 2010 to 2020.

Annual incidence of human and livestock anthrax (per 10,000), human mortality (per 10,000), and annual provincial-level livestock anthrax vaccine coverage (percent) were calculated and graphed in Microsoft Excel for comparing incidence with vaccine coverage. Districts were categorized by vaccine coverage and mapped in QGIS (Supplementary Data S1).

Commune-level crude cumulative incidence (CI; per 10,000) was calculated for humans and livestock. Next, CI rates were smoothed using Spatial Bayes Smoothing (SBS) and Empirical Bayes Smoothing (EBS) in GeoDa version 1.20 (Anselin et al., 2006) (Supplementary Data S1). Spatial smoothing aims to stabilize the variability of CI caused by variation in the number of cases and population at risk. In this study, SBS CI reduced outliers in rate better than EBS (Supplementary Figs. S1 and S2). Commune-level SBS CI were mapped in QGIS using shapefiles from GADM version 3.6.

### Ethics statement

This study underwent ethical review by the Institutional Review Board of National Institute of Hygiene and Epidemiology, Vietnam (IRB-VN01057/IORG 0008555; Project IRB certificate number NIHE IRB-03/2020) and the University of Florida (IRB202003189).

## Results

Nineteen human anthrax cases (two deaths) and 50 livestock cases were reported from 2004 to 2020. Figure 1B shows the co-occurrence of anthrax in humans and livestock in 2004, 2005, 2011, and 2017. Provincial level livestock anthrax incidence was highest in 2005 (0.97 per 10,000), then it decreased (0.08 per 10,000) later in the study. Provincial level human anthrax incidence was highest in 2011 (0.16 per 10,000). The most recent human anthrax outbreak was reported in 2017. Human anthrax mortalities occurred in 2011 (0.04 deaths per 10,000).

The province-level annual livestock anthrax vaccine coverage was <6% (Fig. 1B), but it was not uniformly distributed across districts. Higher coverage was reported in districts close to the provincial capital (Cao Bang City; Supplementary Fig. S3). SBS CI was higher in livestock than in humans (up to >80 livestock cases per 10,000 vs. <20 human cases per 10,000). The spatial distribution of SBS CI for humans (Fig. 1C) and livestock (Fig. 1D) illustrates spatial overlap in the northwest and province center.

## Discussion

This study describes the co-occurrence of human and livestock anthrax in Cao Bang province, Vietnam and estimates the spatial distribution of anthrax using spatially smoothed CI from 2004 to 2020. The temporal co-occurrence of human and livestock anthrax was seen in specific years. This is not uncommon for anthrax; animal cases often associate with human outbreaks (Islam et al., 2018). Livestock annual incidence was higher than human incidence (0.97 vs. 0.16 per 10,000 at provincial level for livestock and humans, respectively).

The mismatch in anthrax reporting is periodically reported with single or few animal cases associated with multiple human cases due to meat-sharing practices (Kisaakye et al., 2018). In addition, limited intersectoral data sharing has been noted in other areas (Kracalik et al., 2014). We noted discrepancies in the number of anthrax cases between the reports of provincial human health and animal health sectors before 2010. The discrepancies are indicative for data mismatch/under-reporting and suggest intersectoral data sharing needs strengthening to improve surveillance and control in the province.

The spatial distribution illustrated highest anthrax incidence in the northwest (Bao Lam, Bao Lac districts) and the provincial center (Cao Bang City, Hoa An district). It is reasonable for the anthrax vaccination to be implemented in those districts. The highest vaccine coverage was in Cao Bang City and surrounding districts (Supplementary Fig. S3). This could be due to the smaller size of livestock populations (Supplementary Fig. S4) and the convenience for vaccine deployment (Sarker et al., 2020). However, more study is needed to understand how the province defined priority for geographic areas and the types of livestock vaccinated (buffalo and cattle), and what challenged vaccine deployment in the northwestern area where higher anthrax incidence was reported.

There were study limitations. Discrepancies in data provided by human and animal health sectors could not be verified since the data were not entered into a computerized management system in 2000s. Although vaccination campaign was initiated before 2014, vaccine data were not available from 2004 to 2013 for better comparing vaccine coverage to disease incidences for the whole study period. In addition, we could not retrieve information about case epidemiology for either group.

## Conclusions

Our study indicates temporal and spatial co-occurrence of human and livestock anthrax in Cao Bang province in northernmost Vietnam from 2004 to 2020. Maps of smoothed anthrax incidence can help local authorities better define high-risk areas for implementing livestock anthrax vaccination campaigns and public health education to local communities. Data quality limitations could be addressed by improving data sharing between human and animal health sectors. These implications would be applicable to other provinces sharing similar characteristics with Cao Bang.

## Supplementary Material

Supplemental data
