# Supplementary material for: Incidence and Spatial distribution of Human and Livestock Anthrax in Cao Bang Province, Vietnam (2004–2020)
Source: Vector Borne Zoonotic Dis. 2023 May 12;23(5):306–9. doi: 10.1089/vbz.2022.0072 (PMC10178930; doi:10.1089/vbz.2022.0072)
Supplement: Supplemental data [file Supp_Data.docx]

1. **Materials and Methods**

**The study setting**

Cao Bang situates in the northern Vietnam. It shares border with China in the north and east, Lang Son and Bac Kan provinces in the south, and Ha Giang province in the west (Fig 1A). The province’s administration is organized in provincial, district, and commune level. There are 199 communes divided into 13 districts in the province (Pham, 2020).

**Data collection and management**

The number of human cases, livestock cases and human deaths due to anthrax from 2004-2020 were extracted from the province’s human health and animal health reporting systems by either Cao Bang Center for Disease Control (CDC) or Cao Bang Sub-Department of Plantation and Animal Husbandry (Sub-DAH). The annual numbers of livestock anthrax vaccine doses were available at provincial level from 2014-2020. The province’s disease reporting system was designed to capture human index cases in provincial hospitals, district hospitals, and commune health centers that triggered field outbreak investigations to look for more cases associated with the index cases. In animal health sector, the disease surveillance was conducted through the network of field veterinarians at district and provincial levels. In this study, the anthrax case definition was in accordance with Vietnam’s national disease surveillance system which was any case presenting clinical signs (humans or livestock) and/or symptoms (humans) of anthrax with or without laboratory confirmation as presented in Section 2. Data were aggregated to communes (sub-district polygons) for mapping the spatial distribution of human and livestock anthrax. The commune, district, and provincial polygons were downloaded from GADM data version 3.6 ([*www.gadm.org*](http://www.gadm.org)*).*

**Human and livestock population data**

Human population at commune level for each year from 2004-2020 were estimated in QGIS version 3.24.3 using Zonal statistic routine and gridded unconstrained UN adjusted population counts for individual countries provided by WorldPop database (grid cell resolution is ~100m x 100m at the equator) (<https://www.worldpop.org/>) (Worldpop, 2022).

Similar zonal statistic method was used for estimating livestock population at commune level in 2010 using raster data (resolution of 5 arc minutes) for global distribution of buffalo, cattle, and goat (<https://dataverse.harvard.edu/>) (Gilbert et al., 2018). Livestock referred to buffalo, cattle, and goat in this study since they accounted for the vast majority of livestock and anthrax was only reported among those animals in the province (Sub-DAH provided data).

Additionally, a district-level dataset for livestock population from 2010-2020 was provided by the Department of Animal Health (DAH), Vietnam Ministry of Agriculture and Rural Development. Since the data were not available for 2004-2009, we estimated the livestock population for this period with the assumption that the livestock herd was not significantly different from the numbers in 2010 based on the pattern of livestock population change from 2010-2020.

**Province-level incidence, Livestock anthrax vaccine coverage, Commune-level Cumulative incidence calculation and Spatial smoothing**

$$Province-level Annual incidence (per 10,000)=\frac{Total human/livestock cases in year Y}{Total human/livestock population in year Y} x 10,000 (1)$$

$$Province-level mortality incidence \left( per 10,000 \right)=\frac{Total human deaths in year Y}{Total human population in year Y} x 10,000 (2)$$

$$Province-level vaccine coverage \left( \% \right)=\frac{Total anthrax vaccine doses administered in year Y}{Total buffalo and cattle population in year Y} x 100 (3)$$

In above formulas, *Y* is a given year ranging from 2004 to 2020. Here, zonal estimated human population and DAH provided livestock population at provincial level were used for calculating annual incidence (1), mortality incidence (2), and livestock anthrax vaccine coverage (3). Vaccine coverage was also calculated for each district using the same method. Then, the districts were categorized into district-level vaccine coverage ranges and mapped in QGIS.

Crude Cumulative incidence (CI) of human and livestock anthrax at commune level (per 10,000) were calculated by dividing total number of human/livestock cases in the whole study period (2004-2020) to the median-year populations (4). Here we used zonal estimated populations at commune level for both humans and livestock.

$$Commune-level Crude CI (per 10,000 )=\frac{Total anthrax cases in humans/livestock in commune i}{Human/livestock population of commune i at median year j} x 10,000 (4)$$

In above formula, *i* is a given commune of Cao Bang province, *j* is the median year of the study period (*j*=2012 for humans, *j*=2010 for livestock). Here, 2010 was used as median year for livestock anthrax instead of 2012 because the livestock population data were only available for 2010.

Spatial rate smoothing was performed for human anthrax to stabilize the variability of crude CI caused by variation in numerators (number of cases) and denominators (population at risk). Given that anthrax is a rare disease, it varies largely among the communes, with many communes reporting zero cases while some reported high case numbers. Smoothing was conducted in GeoDa (version 1.20) with two methods including Spatial Bayes Smoothing (SBS) and Empirical Bayes Smoothing (EBS). The smoothing techniques are described elsewhere (Anselin et al., 2006). Both approaches aim to reduce rate variability by adjusting the estimates toward either a global mean (EBS) or local mean defined by a weights matrix (SBS); in both cases, the greatest adjustment is in polygons with low populations. We used a 1^st^ order queen contiguity matrix to define neighbors for the SBS, meaning any neighboring sharing a boundary line or vertex with the commune of interest would be considered a neighbor. The smoothed CI estimates from both methods were plotted using *ggplot* function in R-Studio to illustrate the change after smoothing (RStudio Team, 2020). Although both SBS and EBS methods collapsed the CI means and standard deviation (Fig S1), SBS CI provided a closer estimation while EBS CI overestimated the spatial distribution of anthrax that spread out to the whole province (Fig S2). Commune-level SBS CI were mapped in QGIS using shapefile downloaded from GADM version 3.6 (<https://gadm.org/>).

1. **Clinical signs and symptoms for surveillance of anthrax in humans and livestock**

***2.1 Human anthrax case definition (Decision number 5703/QD-BYT issued by Vietnam Ministry of Health, dated December 20, 2017)*** (Vietnam Ministry of Health, 2017)***:***

- Suspected/clinical case is a person who exposes to animal, animal product that is suspected of animal anthrax or living in endemic area. The acute onset signs and symptoms fall into one of the following categories:
  - Cutaneous anthrax: itchy in the infection site, then it forms small blisters, painless black eschar, normally seen around arm, hand, around the patient’s mouth and knee.
  - Inhalation anthrax: pneumonia-like symptoms but rapid progress to hard breathing and septic shock.
  - Gastrointestinal anthrax: remarkable abdominal pain, fever, and septic shock.
  - Meningitis anthrax: acute onset, seizure, lost consciousness, and other signs and symptoms of meningitis infection.
- Confirmed case is a suspected/clinical case with one of the following confirmatory tests: bacterium identification by culture, or typical genetic material by molecular biological techniques (PCR).

***2.2 Livestock anthrax case definition (Circular number 07/2016/TT-BNNPTNT issued by Vietnam Ministry of Agriculture and Rural Development, dated May 31, 2016)*** (Vietnam Ministry of Agriculture and Rural Development, 2016)***:***

The main signs of anthrax in livestock include tongue exposure, abdominal distention, body fluid containing dark and non-clotting blood run out from mouth, nose, anus, and genital parts. Other signs and symptoms are fever (40^o^C-42.5^o^C), high heartbeat rate, shortened breathing, diarrhea, reduced milk production, abortion, staggering walk, seizure, red eyes. In some cases, the livestock bumps into a bush and dies suddenly or dies after 1-3 days of the onset. Cutaneous symptoms are swollen areas in neck, chest, rump that become cold later, painless, rotten eschar, sometimes forming dark red eschar with yellow fluid. Incubation period in 3-7 days with some exception of 2 days or up to 2 weeks.

1. **Supplemental Figures**

**Fig S1** compares the crude, SBS, and EBS CI that indicates both SBS and EBS collapsed outliers and standard deviation towards the means. **Fig S2** shows the distribution of anthrax using crude, Spatial Bayes Smoothed (SBS), and Empirical Bayes Smoothed (EBS) Cumulative incidence (CI) for human and livestock anthrax in Cao Bang province, Vietnam (2004-2020). Spatial smoothing methods were conducted in GeoDa. It indicates that SBS CI (B – human anthrax, E – livestock anthrax) provides closer estimates compared to Crude CI (A – humans, D – livestock), while EBS CI (C-humans, F-livestock) overestimates the distributions of anthrax for both humans and livestock (the incidence was low but spread out to the whole province). **Fig S3** presents the range of anthrax vaccine coverage for buffalo and cattle at district level from 2014-2020. The vaccine was distributed in northwest districts and districts surrounding Cao Bang City (the province’s capital). The higher percentages were seen in province’s center compared to the northwestern districts. **Fig S4** shows the commune-level distribution of livestock herd in Cao Bang province in 2010. Buffalo, cattle, and goat were more populated in northwestern and southwestern communes.


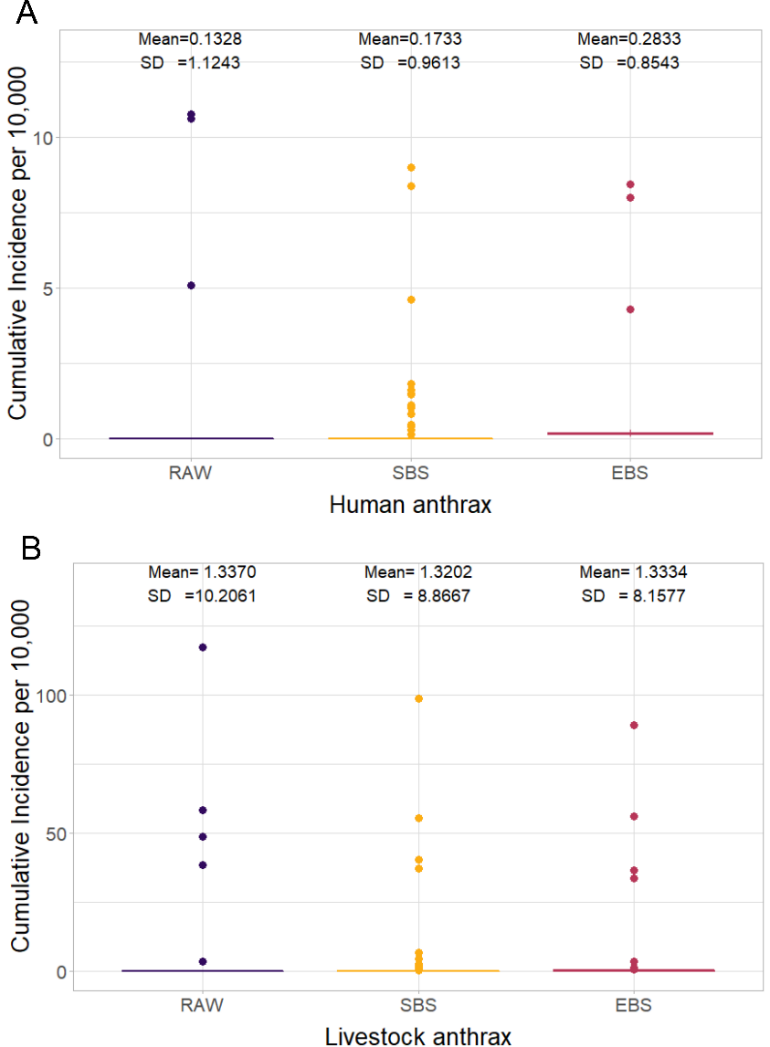


**Fig S1. Comparison of Crude, Spatial Bayes Smoothed (SBS), Empirical Bayes Smoothed (EBS) cumulative incidence for Human anthrax (A) and Livestock anthrax (B) in Cao Bang Province, Vietnam (2004-2020).**


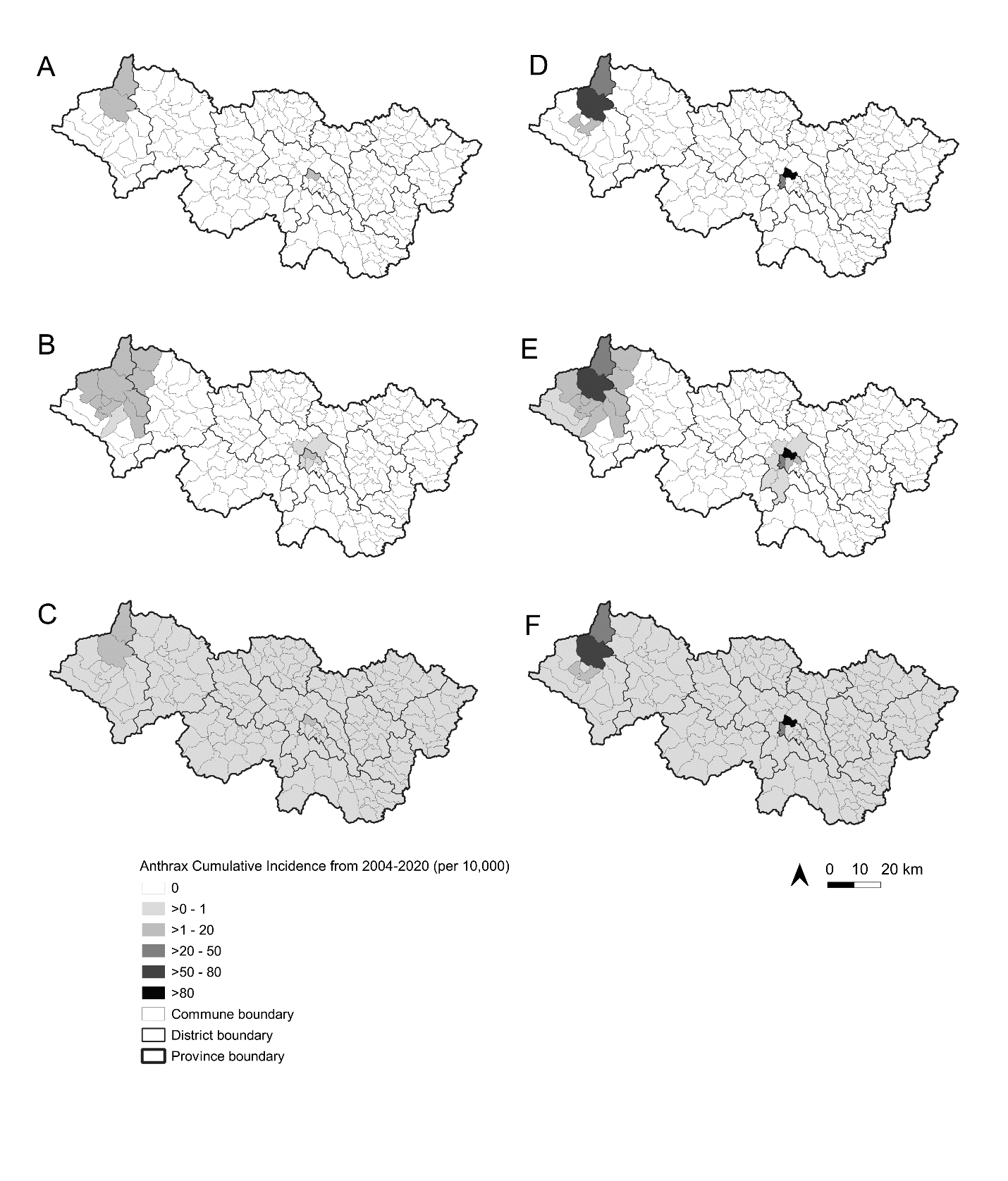


**Fig S2. The distribution of anthrax using Crude, Spatial Bayes Smoothed (SBS), and Empirical Bayes Smoothed (EBS) Cumulative incidence (CI) for humans (A - Crude CI, B-SBS CI, C-EBS CI), and livestock (D - Crude CI, E-SBS CI, F-EBS CI) in Cao Bang province, Vietnam (2004-2020).** Spatial smoothing methods were conducted in GeoDa.


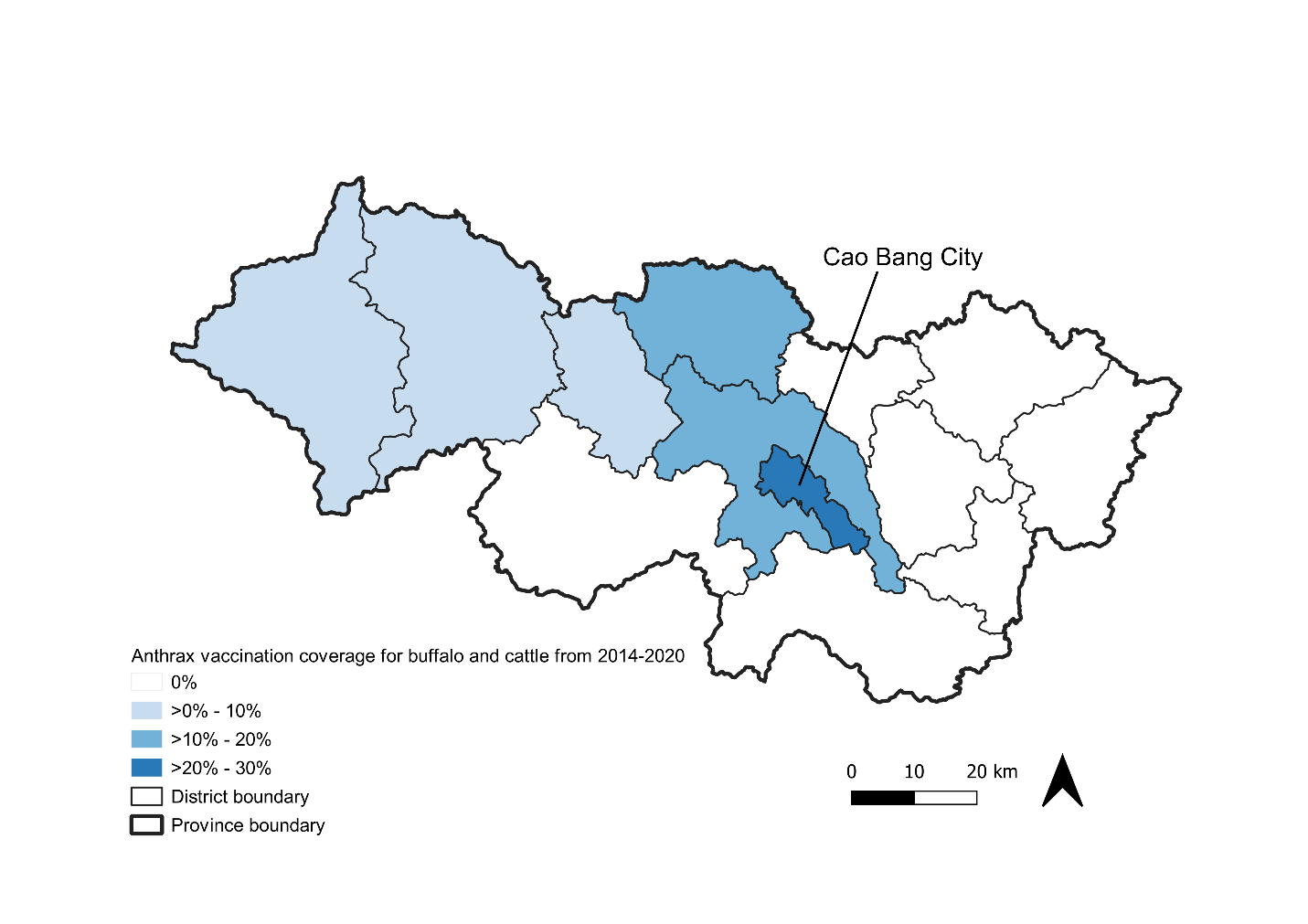


**Fig S3. District-level annual livestock anthrax vaccine coverage from 2014-2020 (buffalo and cattle) in Cao Bang Province, Vietnam**


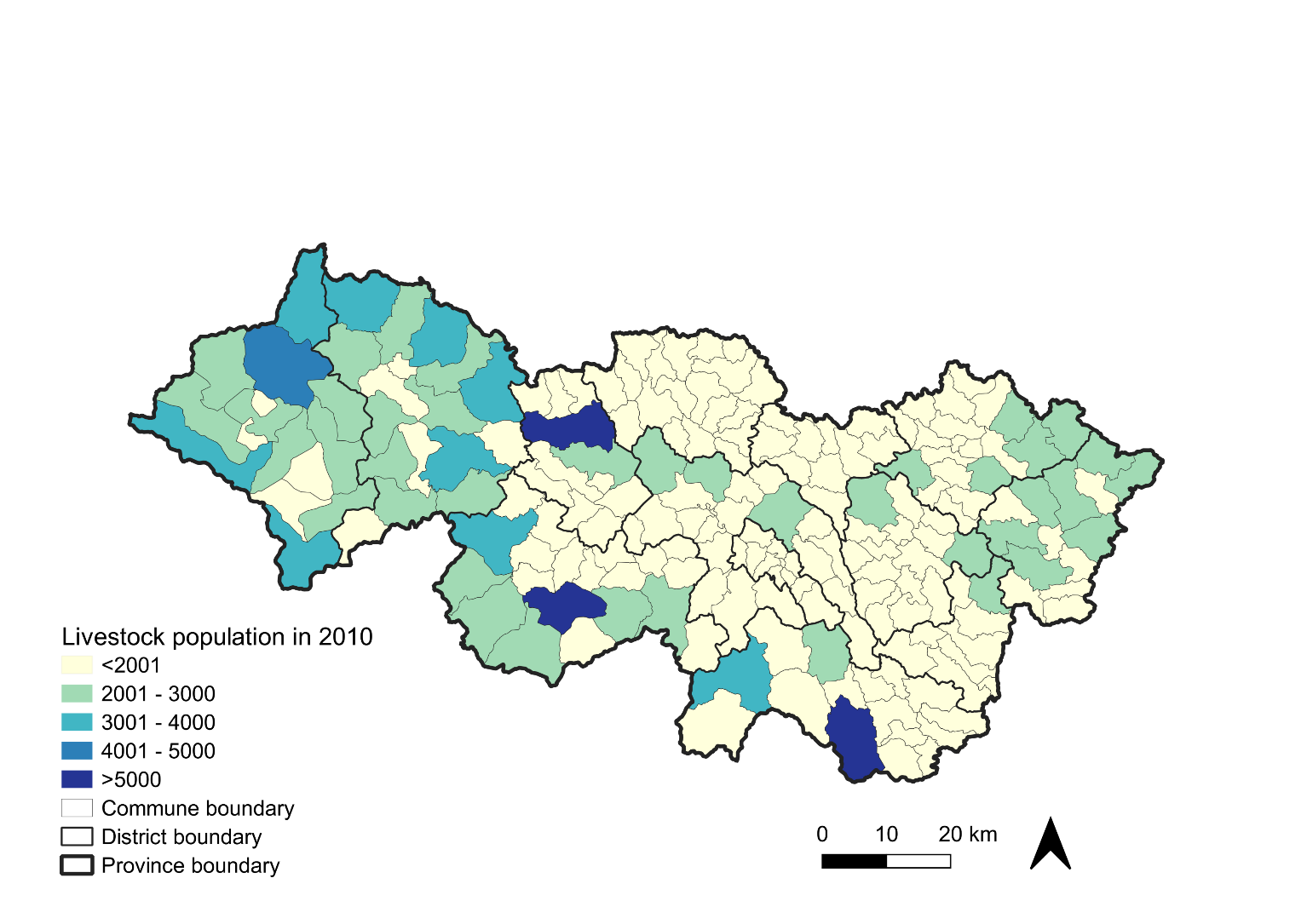


**Fig S4. Choropleth map of commune-level livestock population in 2010 (buffalo, cattle, and goat) in Cao Bang Province, Vietnam.**

**References**

Anselin L, Lozano N and Koschinsky J. Rate Transformation and Smoothing. University of Illinois; 2006.

Gilbert M, Nicolas G, Cinardi G, et al. Global Distribution Data for Cattle, Buffaloes, Horses, Sheep, Goats, Pigs, Chickens and Ducks in 2010. Sci Data 2018;5(1):180227; doi: 10.1038/sdata.2018.227.

Pham TP. Cao Bang Statistical Yearbook 2019. Cao Bang Statistic Office: Vietnam; 2020.

RStudio Team. RStudio: Integrated Development for R. 2020.

Vietnam Ministry of Agriculture and Rural Development. Circular Number 07/2016/TT-BNNPTNT Regulating the Prevention and Control of Diseases in Domestic Land Animals. 2016. Available from: https://thuvienphapluat.vn/van-ban/The-thao-Y-te/Thong-tu-07-2016-TT-BNNPTNT-phong-chong-dich-benh-dong-vat-tren-can-313499.aspx [Last accessed: 11/5/2021].

Vietnam Ministry of Health. Decision Number 5703/QĐ-BYT 2017 Issuing the Guideline for Surveillance, Prevention, and Control of Anthrax in Human. 2017. Available from: https://thuvienphapluat.vn/van-ban/The-thao-Y-te/Quyet-dinh-5703-QD-BYT-2017-Huong-dan-giam-sat-va-phong-chong-benh-than-tren-nguoi-431825.aspx [Last accessed: 11/5/2021].

Worldpop. Population Counts: Unconstrained Individual Countries 2000-2020 UN Adjusted (100m Resolution). 2022. Available from: https://www.worldpop.org/geodata/listing?id=69.
